# Evaluation of Tellurium as a Fuel Additive in Neodymium-Containing U-Zr Metallic Fuel

**DOI:** 10.1038/s41598-019-51852-z

**Published:** 2019-11-05

**Authors:** Nathan D. Jerred, Rabi Khanal, Michael T. Benson, Emmanuel Perez, James A. King, Megha Dubey, Jatuporn Burns, Indrajit Charit, Samrat Choudhury, Robert D. Mariani

**Affiliations:** 1 Department of Chemical and Materials Engineering, University of Idaho, Moscow, Idaho 83844 USA; 2 Materials and Fuels Complex Division, Idaho National Laboratory, Idaho Falls, Idaho 83415 USA; 3 Microscopy and Characterization Suite, Center for Advanced Energy Studies, Idaho Falls, Idaho 83401 USA; 4 Nuclear Science and Technology Division, Idaho National Laboratory, Idaho Falls, Idaho 83415 USA

**Keywords:** Energy science and technology, Engineering, Materials science

## Abstract

Phase-stability in a U-Zr-Te-Nd multi-component metallic fuel for advanced nuclear reactors is systematically investigated by taking into account binary, ternary and quaternary interactions between elements involved. Historically, the onset of fuel-cladding chemical interactions (FCCI) greatly limits the burnup potential of U-Zr fuels primarily due to interactions between lanthanide fission products and cladding constituents. Tellurium (Te) is evaluated as a potential additive for U-Zr fuels to bind with lanthanide fission products, e.g. neodymium (Nd), negating or mitigating the FCCI effect. Potential fresh fuel alloy compositions with the Te additive, U-Zr-Te, are characterized. Te is found to completely bind with Zr within the U-Zr matrix. Alloys simulating the formation of the lanthanide element Nd within U-Zr-Te are also evaluated, where the Te-Nd binary interaction dominates and NdTe is found to form as a high temperature stable compound. The experimental observations agree well with the trends obtained from density functional theory calculations. According to the calculated enthalpy of mixing, Zr-Te compound formation is favored in the U-Zr-Te alloy whereas NdTe compound formation is favored in the U-Zr-Te-Nd alloy. Further, the calculated charge density distribution and density of states provide sound understanding of the mutual chemical interactions between elements and phase-stability within the multi-component fuel.

## Introduction

Phase stability in a multi-component alloy is determined by a complex set of mutual chemical, elastic and other energetic interactions between elements of the alloy. Such binary, ternary, and quaternary interactions between two or more elements lead to the minimization of the total energy of the alloy. Understanding such interactions is critical to the design of alloy chemistry for a targeted application. In this study, experimental and theoretical efforts are combined to understand such mutual interactions between elements to investigate the overall phase stability in a U-Zr-Te-Nd multi-component alloy. The binary U-Zr metallic nuclear fuels have largely been used in fast-spectrum reactors, such as the Experimental Breeder Reactor II (EBR-II), and they continue to be attractive for advanced nuclear reactor concepts, such as the advanced sodium-cooled fast reactor (SFR). Some major advantages of such fuel systems are their ease of fabrication and reprocessing, high thermal conductivity, good compatibility with the sodium coolant, and their ability to contain high densities of fissile and fertile materials^1–3^. However, the onset of chemical interactions between fission products and fuel components with cladding constituents greatly limits the burnup potential of U-Zr fuels.

The lanthanide elements have been found to be a major cause of chemical interactions at the fuel-cladding interface, also known as fuel-cladding chemical interaction (FCCI). Such lanthanides form in the fuel as fission products and tend to migrate to the fuel periphery; being primarily driven by temperature gradients via ‘liquid-like’ transport^4–6^. Metallic fuels tend to swell due to fission gas formations within the first 1–2 at.% burnup leading to intimate contact to be made between the fuel and cladding components^2,7^. With prolonged reactor operation, such fuel – cladding contact leads to the initiation of FCCI. Lanthanide elements (Nd, Ce, La and Pr), being primarily immiscible in the U-Zr system, along with fuel constituents tend to interact with cladding constituents at the fuel – cladding interface. Such interactions can lead to the formation of brittle, low-melting phases exacerbating cladding wastage, which can eventually lead to cladding breach^4^. Therefore, to increase metallic fuel longevity and achieve greater fuel burnups, an effective method to mitigate FCCI is needed.

Several methods have been proposed to control FCCI, including internal cladding liners^8^, internal cladding coatings^9^ and fuel additives^6^. As opposed to acting as a diffusion barrier, i.e. liners and coatings, fuel additives are alloyed with the fuel to form high-temperature, stable compounds with lanthanide fission products as they form within the fuel during irradiation, stabilizing them within the fuel meat and limiting their interaction with cladding constituents at the fuel – cladding interface. Criteria for potential additives have previously been developed by Mariani *et al*.^6^, several of which have been studied – Pd^6,10^, Sn^11^, Sb^12,13^, Sn + Sb^14^, and In^13,15^, and have demonstrated additives as a promising approach to counter FCCI. Additionally, As and Se have been identified as potential additives based on density functional theory (DFT) calculations and experimental observations^16^.

Tellurium (Te) was initially identified as a potential additive for metallic nuclear fuels by Mariani *et al*.^6^ but detailed experimental work has been limited in this area. The focus of the present study is an evaluation of Te’s effectiveness to bind with neodymium (Nd). First principles studies carried out by Khanal *et al*.^16^ show agreement in the potential of Te to bind with Nd in an α-U matrix. Recent research conducted by Xie *et al*.^17^ characterized the interactions of Te with another lanthanide element, cerium (Ce), in a U-10Zr alloy. In their evaluation of the U-10Zr-Te alloy, they were unable to obtain direct evidence of the formation of a Zr-Te phase; instead they postulated the formation of a Te-Zr-U ternary phase. Interestingly, the current research confirms the formation of a non-equilibrium phase Zr_2_Te in a similar alloy composition. Furthermore, it is worth noting that in their characterization of the U-10Zr-Te-Ce alloy, their results indicate Te and Ce primarily form the stable compound CeTe.

In the present study, the microstructure of potential fuel compositions that may be resistant to FCCI are characterized, followed by a characterization of fuel compositions that contain Nd to simulate the formation of lanthanide fission products through fuel burnup. Nd was chosen for this study due to its identification as a predominant lanthanide to form within U-10Zr fuel irradiated in a liquid breeder fast reactor EBR-II^6^ and has been observed to readily diffuse into the cladding^4^. Furthermore, this research focuses on the mutual interactions between elements in the U-Zr-Te and U-Zr-Te-Nd multi-component alloys, which lead to stable phase formations within the U-Zr matrix.

## Results and Discussion

Four alloys evaluated in the current work are listed in Table 1. The 2Te and 4Te alloys represent potential fresh U-Zr fuels containing Te as an additive. The 2Te-2Nd and 4Te-4Nd alloys represent simplified fuel compositions that simulate the formation of lanthanide fission products within the fuel through irradiation, where Nd represents the lanthanide fission product. The Te addition is based on the anticipated amount needed to bind all of the lanthanides that form in the fuel at the respective concentrations. The concentration of neodymium in the alloys represents the anticipated total lanthanide amount to form within the fuel at both 8 and 16 at.% burnups based on fission product concentrations reported by Mariani *et al*.^6^. Essentially, the additive – lanthanide atomic ratio is targeted to be 1:1; however, the alloys are in essence slightly additive-rich to counter possible volatilization loss of tellurium during casting.

**Table 1:** Alloys evaluated in this study.

Alloy	Composition in wt.%	Composition in at.%
2Te	U-10Zr-2Te	U-22.2Zr-3.17Te
4Te	U-10Zr-4Te	U-21.8Zr-6.24Te
2Te-2Nd	U-10Zr-2.16Te-2Nd	U-21.9Zr-3.4Te-2.8Nd
4Te-4Nd	U-10Zr-4.3Te-4Nd	U-21.3Zr-6.6Te-5.4Nd

During initial arc-melting and subsequent drop casting of the alloys, some loss of Te was observed. Tellurium is considered a metalloid and has a relatively low melting temperature of 723 K (450 °C) and boiling point of 1263 K (990 °C), which leads to a narrow temperature range where the element can remain in a liquid state. Loss of Te primarily occurred during its addition to the U-10Zr (wt.%) button, which requires an increased amount of energy to fully liquefy having a solidus temperature of approximately 1521 K (1248 °C) and liquidus temperature between 1628 K–1633 K (1355 °C–1360 °C)^18^. Once Te was homogenized into the U-10Zr (wt.%) alloy, it became stabilized by the formation of Zr-Te precipitates (further discussed in later sections).

### Microstructural and compositional analysis of U-10Zr with Te additive

**Figure 1:**
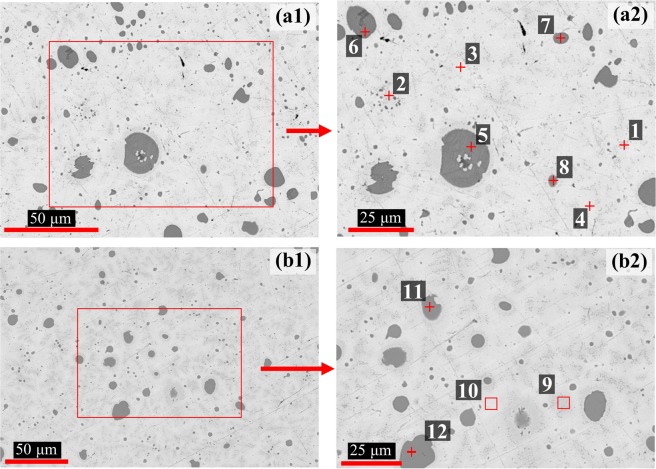
BSE SEM images of (**a1**) the 2Te alloy and (**b1**) the 4Te alloy; (**a2**,**b2**) are the magnified BSE SEM images of the ‘red’ rectangular inset regions in (**a1**) and (**b1**), respectively.

The microstructures of the 2Te and 4Te alloys are shown in the scanning electron microscopy (SEM) backscatter electron (BSE) images in Fig. 1. Round grey-contrast precipitates are observed within the matrix of both alloys ranging in size from sub-micron to approximately 20 μm across. It should be noted that the matrix, a combination of a primary light phase with a dispersed light-grey phase, is the expected phase formation of the U-Zr system in the as-cast condition. Extensive work has been done on U-Zr alloys, and in particular the U-10Zr (wt.%)^18–24^. Furthermore, the phase formations between U and Zr in the as-cast condition, specifically the co-formation of the α-U and δ-UZr_2_ phases, have been discussed elsewhere^19,23^. A more recent study by Irukuvarghula *et al*.^24^ concluded the phase formations for the majority of U-Zr alloys, including the U-10Zr alloy, in the as-cast condition are likely α-U phase and a disordered, metastable ω phase. Even though the ω and δ phases have the same chemical composition, the δ phase is partially ordered. Given the extensive knowledge base on the U-Zr binary system, the present study focuses on the observed ternary interactions of Te, U, and Zr and how the addition of Nd changes such mutual interactions.

**Figure 2:**
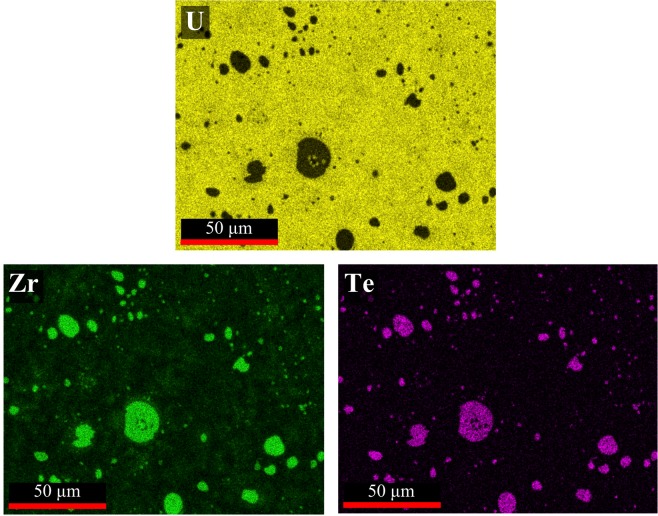
Elemental EDS maps of the 2Te alloy. Analysis location aligns with the area imaged in Fig. 1(a1).

Elemental maps of U, Zr and Te, as obtained from energy dispersive X-ray spectroscopy (EDS) studies of the 2Te alloy for the location displayed in Fig. 1(a1), are shown in Fig. 2. The maps reveal that the precipitates are the combination of Zr and Te contained within a primarily U matrix. Elemental compositions of areas designated in Fig. 1(a2,b2) are listed in Table 2. Based on EDS point analysis, the precipitates (points 5–8, 11, 12) exhibit a ratio range of 2:1 to 2.4:1 (Zr:Te), indicating that they are likely the Zr_2_Te compound. Small white inclusions are also detected in the larger spherical precipitates (Fig. 1(a1)). Such inclusions are likely trapped matrix material. Xie *et al*. also made similar observations in their analysis of a U-10Zr-Te alloy^17^.

**Table 2:** SEM EDS elemental data results of analysis points depicted in Fig. 1 (at.%).

	Fig. 1(a2)					Fig. 1(b2)			
	U	Zr	Te	Phase^a^		U	Zr	Te	Phase^a^
1	62	31	7	U-Zr^b^	*9*	59	40	2	U-Zr^b^
2	60	38	2	U-Zr^b^	10	83	16	1	U-Zr^b^
3	84	14	2	U-Zr^b^	11	6	67	27	Zr_2_Te
4	77	21	2	U-Zr^b^	12	5	67	28	Zr_2_Te
5	9	64	27	Zr_2_Te					
6	6	67	27	Zr_2_Te					
7	7	62	31	Zr_2_Te					
8	11	63	26	Zr_2_Te					

^a^Suggested phase based on EDS analysis.

^b^Matrix phase of the as-cast U-Zr alloy.

**Figure 3:**
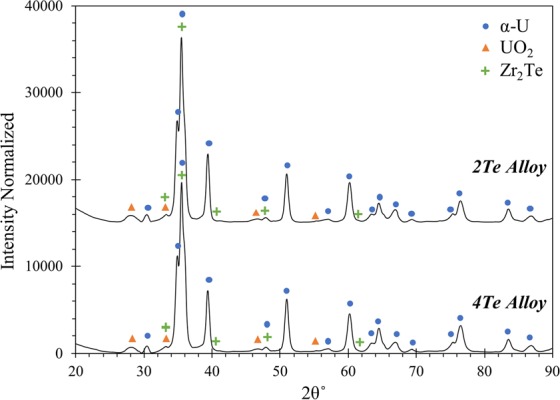
XRD patterns of the 2Te and 4Te alloys.

Focused X-ray diffraction (XRD) analyses of alloys 2Te and 4Te alloys are presented in the form of XRD patterns in Fig. 3, and the corresponding crystallographic information listed in Table 3. The XRD pattern shows that the alloys are primarily comprised of the α-U based solid solution phase. The presence of UO_2_ was also seen and its formation is likely due to the specimen exposure to air during analysis. The Zr_2_Te compound was also detected confirming the EDS elemental data obtained on the precipitates in the alloy. The Zr_2_Te phase is not depicted as an equilibrium phase on the Zr-Te binary phase diagram by Okamoto^25^. However, more recent work by Örlygsson and Harbrecht^26^ detailed the existence of the Zr_2_Te phase. Their study detected the Zr_2_Te phase in an as-cast specimen using EDS analysis and powder-based XRD. Further, they suggested that the Zr_2_Te phase is formed through a peritectic reaction near the incongruent melting point of the Zr_3_Te phase. It is plausible that Zr_2_Te is a metastable phase, which may decompose to stable Zr_5_Te_4_ and Zr_3_Te compounds^25^.

**Table 3:** Crystallographic information for all phases indexed through XRD analysis.

Compound	Pearson Symbol	Space Group	Cell parameters	Alloy ID^a^			
				2Te	4Te	2Te-2Nd	4Te-4Nd
α-U^31^	oS4	Cmcm (63)	a = 0.286, b = 0.585, c = 0.498 [nm] α = β = γ = 90°	x	x	x	x
UO_2_ ^36^	cF12	Fm $\bar{3}$ m (225)	a = b = c = 0.547 [nm] α = β = γ = 90°	x	x	x	x
Zr_2_Te^26^	oP36	Pnma (62)	a = 1.995, b = 0.382, c = 1.065 [nm] α = β = γ = 90°	x	x		
Zr_3_Te^37^	tI32	I $\bar{4}$ (82)	a = b = 1.134, c = 0.563 [nm] α = β = γ = 90°				x
NdTe^38^	cF8	Fm $\bar{3}$ m (225)	a = b = c = 0.627 [nm] α = β = γ = 90°			x	x
Nd_2_O_3_ ^39^	cI80	Ia $\bar{3}$ (206)	a = b = c = 1.110 [nm] α = β = γ = 90°			x	x
Au^40^	cF4	Fm $\bar{3}$ m (225)	a = b = c = 0.408 [nm] α = β = γ = 90°			x	x

^a^Denotes the alloy in which the particular phase was detected through XRD analysis.

In analyzing U-10Zr-Te alloys, it is important to note that no free Te was observed within the alloys; instead it is clear that Te is primarily binding with Zr to form precipitates. Similar observations have been made in other additives-based research in U-10Zr matrix^11,12,15,17^. Such Zr-Te phase formation is deemed beneficial as they would stabilize the additive within the fresh fuel alloy. Here the precipitates were found to primarily consist of the Zr_2_Te phase, which implies that the fuel composition, i.e. U-10Zr, is not being maintained when Te is added to the fuel. As Zr is added to increase the solidus temperature of the alloy, the loss of Zr from the matrix solid solution may decrease the overall solidus temperature of the fuel matrix. To counter such outcome, additional Zr may be needed to maintain the necessary solidus temperature when additives are employed.

### Microstructural and compositional analysis of U-10Zr-2Te-2Nd and U-10Zr-4Te-4Nd alloys

To evaluate the effectiveness of the Te additive to bind with potential lanthanide fission products, Nd was added into the fuel alloys. Here two such alloys listed in Table 1 were evaluated. The Te addition of 2.16 wt.% and 4.3 wt.% corresponds to a targeted 1:1 atomic ratio of additive to lanthanide at the respective burnups. It is important to note that Nd was added to a U-Zr-Te alloy, and therefore the microstructure of the pre-alloy can be presumed to be similar to the previously discussed U-Zr-Te alloys. Therefore, in order for the additive (Te) to bind with the lanthanide (Nd), the Zr_2_Te precipitates would need to decompose. As previously mentioned, in casting Nd into the U-Zr-Te alloy, the Te is stabilized by the formation of Zr_2_Te. Based on the research of Örlygsson and Harbrecht^26^ it can be inferred that Zr_2_Te has a melting temperature sufficiently higher than the melting temperature of Nd (*T*_melt_ = 1021 °C), therefore loss of Te was not likely when Nd was added.

**Figure 4:**
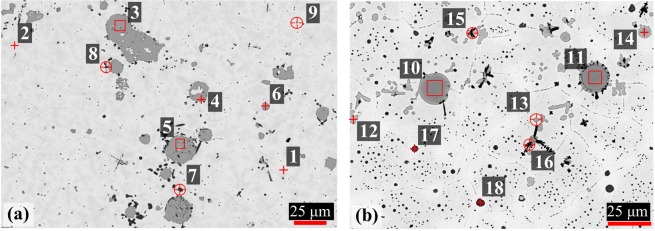
BSE images of (**a**) the 2Te-2Nd and (**b**) the 4Te-4Nd alloys.

Representative optical micrographs of the 2Te-2Nd and 4Te-4Nd alloys are shown in the SEM-BSE images of Fig. 4(a,b), respectively. Both alloys have multiple precipitated phases within the U-Zr matrix. Both show a combination of larger grey-contrast precipitates (approximately 25 µm in size) along with various sized, smaller grey-contrast precipitates. Furthermore, dendritic and small, round black precipitates are observed to form throughout the matrix, with instances of the latter precipitates forming around the large grey-contrast precipitates. Both alloys exhibit the light- and light-grey matrix phases of the U-Zr as-cast structure as previously observed in the U-Zr-Te alloys. The primary difference between the two U-Zr-Te-Nd alloys is the presence of small, round black precipitate clusters that have formed within sections of the light-grey matrix in the 4Te-4Nd alloy (Fig. 4(b)).

**Figure 5:**
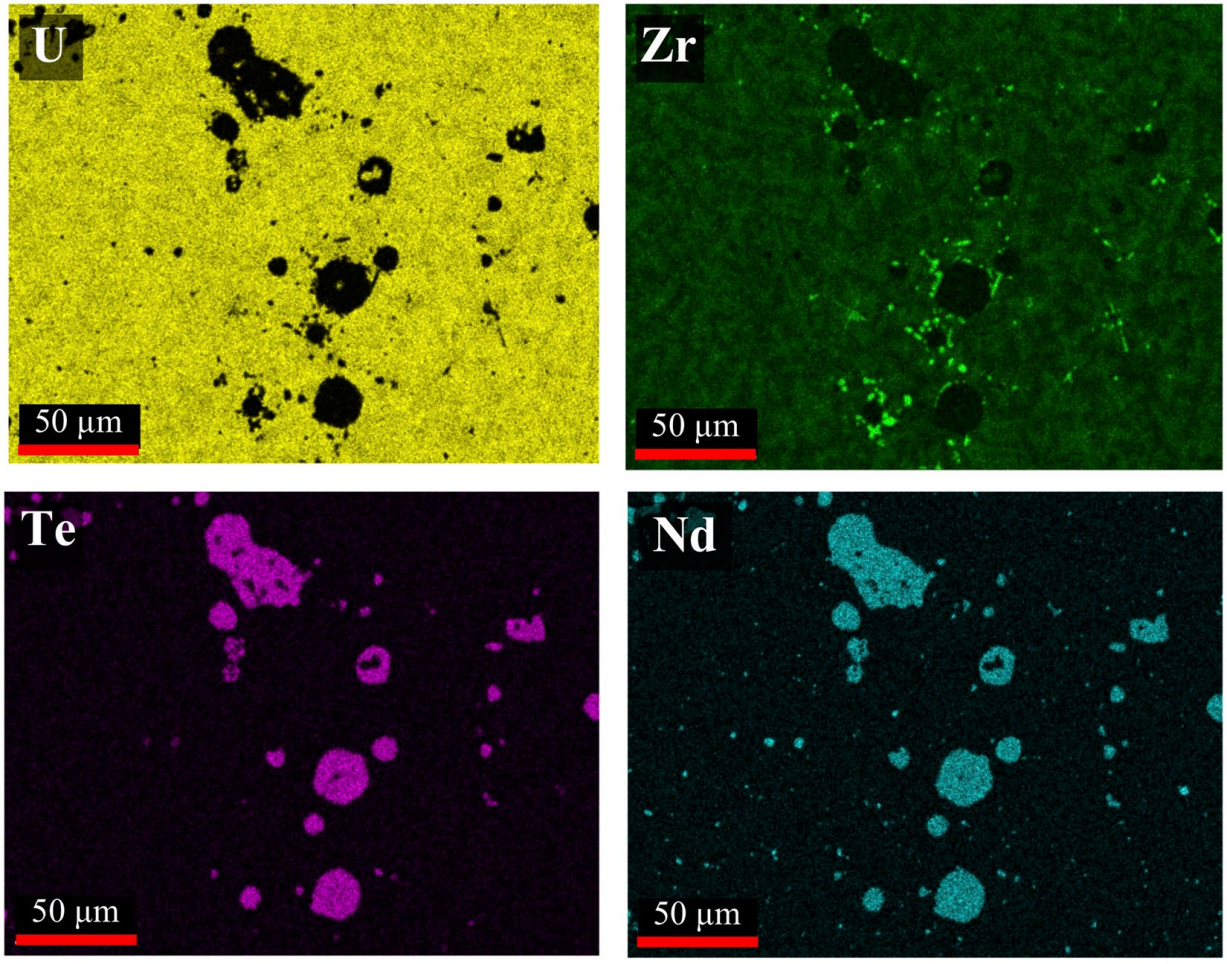
Elemental EDS maps of the 2Te-2Nd alloy. Analysis location aligns with the area imaged in Fig. 4(a).

**Figure 6:**
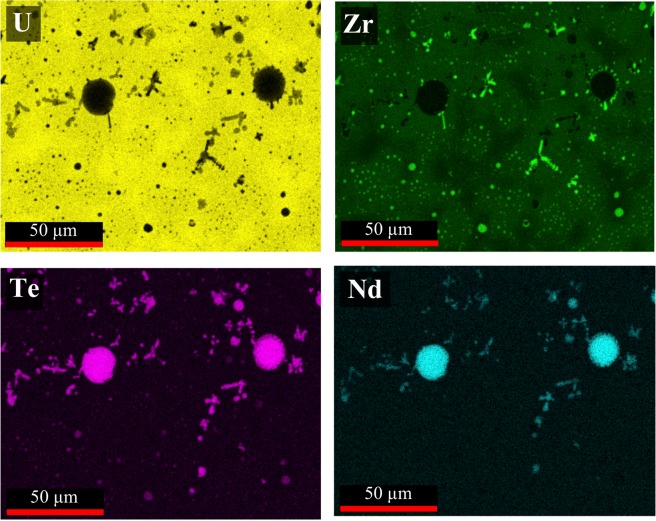
Elemental EDS X-ray maps of the 4Te-4Nd alloy. Analysis location aligns with the area imaged in Fig. 4(b).

Elemental EDS maps for the 2Te-2Nd and 4Te-4Nd alloys, which correspond to the locations imaged in Fig. 4, are shown in Figs 5 and 6, respectively. The EDS maps in Fig. 5 show that the larger grey-contrast precipitates observed in the 2Te-2Nd alloy consist of Te and Nd, whereas the small black and grey-contrast precipitates are found to primarily contain Zr and Nd, respectively. In the 2Te-2Nd alloy, the Te appears to be contained in all of the same precipitates as Nd and no longer appears to be contained with any Zr-containing precipitates. The EDS maps in Fig. 6 show that the grey-contrast precipitates and the dendritic precipitates observed in the 4Te-4Nd alloy also consist of Te and Nd, and Zr, respectively. However, the round black clustered precipitates are found to be primarily Zr with several containing Te. Furthermore, there does not appear to be precipitates that contain only Nd.

EDS point analysis conducted on various precipitates in each alloy is tabulated in Table 4 based on locations indicated in Fig. 4(a,b). The elemental composition of the grey-contrast precipitates, depicted by points 3–6, 10 and 11, indicates that they are likely the NdTe compounds. Analysis of the smaller grey-contrast precipitates in the 4Te-4Nd alloy (depicted by points 12–14) indicates that they are more Te-rich than the NdTe (1:1) compound but also contain appreciable amounts of U (20 at.%, 12 at.%, and 14 at.%). Given the small size of the precipitates, some of the U detected could be from the alloy matrix due to the interaction volume of the electron beam. These Te-rich Nd-Te phases could either have uranium dissolved within or be the formation of a ternary U-Te-Nd phase. To resolve the exact nature of the precipitate, higher resolution characterization via transmission electron microscopy (TEM) was carried out on similar precipitates from the 4Te-4Nd alloy, which is discussed in detail below. Analysis of the very small (approximately 1–2 *μm*) grey-contrast precipitates (point 9) in the 2Te-2Nd alloy were found to primarily consist of Nd. The presence of pure Nd inclusions indicates the Nd concentration is in excess to that of Te within the alloy. This is further confirmed because no Zr-Te containing compound phases were observed in the alloy and therefore all the Te available bound with the Nd.

**Table 4:** SEM EDS elemental data results of analysis points depicted in Fig. 4(a,b) (in at.%).

	Fig. 4(a)						Fig. 4(b)				
	U	Zr	Te	Nd	Phase^a^		U	Zr	Te	Nd	Phase^a^
1	75	22	2	1	*U-Zr* ^*b*^	10	1	3	50	46	*NdTe*
2	57	42	0	1	*U-Zr* ^*b*^	11	1	4	50	46	*NdTe*
3	0	6	45	49	*NdTe*	12	20	4	56	21	*U-Te-Nd*
4	0	6	45	48	*NdTe*	13	12	3	53	32	*U-Te-Nd*
5	1	7	44	47	*NdTe*	14	14	4	54	28	*U-Te-Nd*
6	0	6	46	48	*NdTe*	15	3	91	6	0	*Zr*
7	1	99	0	0	*Zr*	16	7	93	1	0	*Zr*
8	4	95	0	1	*Zr*	17	3	74	23	0	*Zr*_3_ *Te*
9	5	11	0	84	*Nd*	18	3	75	22	0	*Zr*_3_ *Te*

^a^Suggested phase based on EDS analysis.

^b^Matrix phase of the as-cast U-Zr alloy.

The small, dendritic black precipitates observed in both alloys are found to consist of high concentrations of Zr (points 7, 8, 15, and 16 in Fig. 4). Such Zr-rich phases were not expected within the alloy. However, Janney and O’Halleran^27^ observed similar Zr-rich phases in an as-cast structure of U-Zr with actinide and lanthanide additions. They found that the Zr-rich precipitates tend to form near lanthanide-containing precipitates, similar to what was observed here. They also suggested that such Zr-rich precipitates might get stabilized during casting and subsequent cooling due to trace impurities of C, N and O.

Evaluation of the larger round black precipitates (approximately 4–5 *μm*) within the clusters (points 17 and 18 in Fig. 4b) in the 4Te-4Nd alloy indicates that they may be Zr_3_Te compound; a more Zr-rich compound than the Zr_2_Te phases previously observed in the U-Zr-Te alloys. It should also be noted that points 17 and 18 indicate a higher Zr concentration than what would be expected in the Zr_3_Te compound. Given the size of the Zr-Te precipitates and the fact that they are clustered within a Zr-rich region of the U-Zr matrix, the higher Zr content and presence of U are most likely due to the beam interactions with the surrounding matrix. Because of the small size (submicron) of the clustered black precipitates, their accurate elemental composition could not be reliably assessed via SEM equipment used in this study.

**Figure 7:**
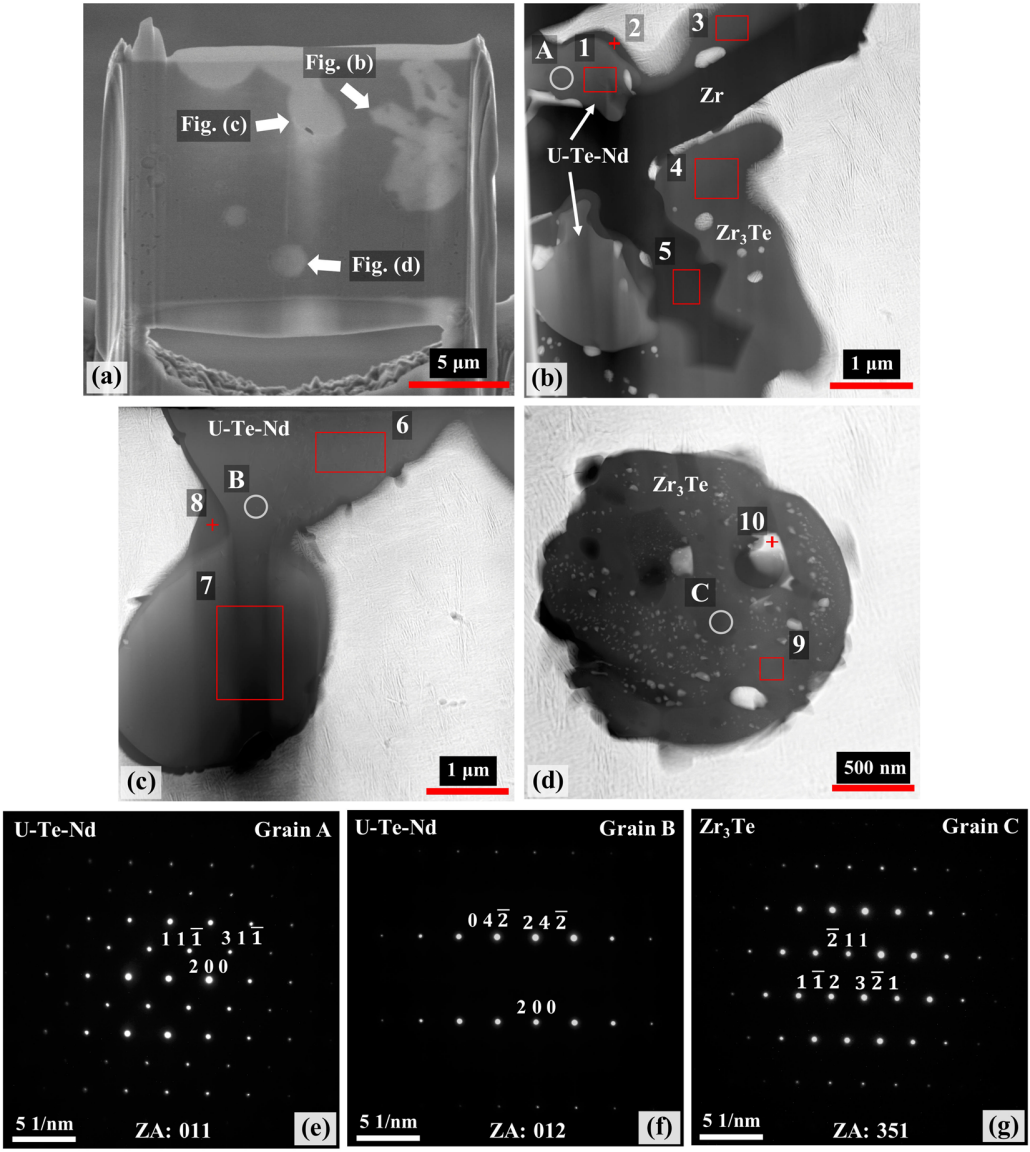
TEM analysis results of precipitates in the 4Te-4Nd alloy. (**a**) SEM image of the TEM lamella, and HAADF micrographs of (**b**) U-Te-Nd, Zr-Te, and Zr precipitates; (**c**) U-Te-Nd precipitate; and (**d**) Zr-Te precipitate. Single zone-axis SAED patterns from (**e**) grain *A* along the [011] zone axis, (**f**) grain *B* along the [012] zone axis, and (**g**) grain *C* along the [351] zone axis.

To positively identify the phases of the smaller precipitates previously observed in the 4Te-4Nd alloy, TEM analysis was performed. The TEM specimen, shown in Fig. 7(a), was made using a focused ion-beam (FIB) lift-off technique targeting a similar precipitate to that observed between points 12 and 14 in Fig. 4(b). The fabricated TEM specimen included a Zr-Te precipitate similar to that at point 16. High-angle annular dark field (HAADF) images and selected area electron diffraction (SAED) patterns of the precipitates are presented in Fig. 7. SAED patterns were collected using multiple zone-axes of a single grain within the phase of interest. A SAED pattern from each analysis area is shown in Fig. 7(e–g). The location of the grains used to generate SAED patterns are denoted by circles in the HAADF images of Fig. 7(b–d). EDS analysis was also performed on the TEM specimen to determine the elemental concentrations of the various precipitates. The analysis areas are denoted in the HAADF images of Fig. 7(b–d); the results are listed in Table 5.

**Table 5:** TEM EDS elemental data results (in at%) of analysis points depicted in Fig. 7(b–d).

	Fig. 7(b)						Fig. 7(c,d)				
	U	Zr	Te	Nd	Phase^a^		U	Zr	Te	Nd	Phase^a^
1	42.1	2.3	42.8	12.9	*U-Te-Nd*	6	38.1	0.9	39.6	21.3	*U-Te-Nd*
2	7.1	78.5	14.4	0	*Zr*_3_ *Te*	7	40.7	0	44	15.3	*U-Te-Nd*
3	3.4	77.6	18.8	0.2	*Zr*_3_ *Te*	8	1.7	75.4	21.5	1.5	*Zr*_3_ *Te*
4	4.0	76.5	19.4	0.1	*Zr*_3_ *Te*	9	3.0	76.5	19.2	1.4	*Zr*_3_ *Te*
5	0.3	99.6	0.1	0	*Zr*	10	83.2	16.3	0.3	0.2	*U-Zr* ^*b*^

^a^Suggested phase based on EDS analysis.

^b^Matrix phase of the as-cast U-Zr alloy.

The HAADF micrographs shown in Fig. 7(b–d) exhibit different types of precipitates. Elemental analysis of the lighter grey-contrast phases, depicted by points 1, 6 and 7, indicates that they are the combination of Nd, Te and U; similar to points 12–14 in Table 4. SAED patterns, obtained from locations *A* and *B* in Fig. 7(b,c) respectively, are both found to index with the NdTe and UTe compounds, both of which are cubic and have similar crystallographic characteristics (Table 6). The formation of UTe precipitates within an alloy containing both Zr and Nd is not likely based on the thermodynamic characteristics of U-Te, Zr-Te and Nd-Te; as discussed further in the computational section later. Other potential binary compounds were considered – Nd_2_Te_3_, NdTe_2_, Nd_2_Te_5_, and NdTe_3_ – albeit none were found to match with the SAED patterns of a given grain. The few U-Nd-Te ternary compounds noted in literature were considered – UTe_4_Nd_2_, UTe_5_Nd_2_, and UTe_6_Nd – but none could be indexed with the SAED patterns. The existence of stable U-Te-Nd compounds indicates that the formation of such a ternary phase is possible. It is worth noting that the U-Te-Nd phases documented in literature are Te-rich, which is a similar scenario to those precipitates analyzed in Fig. 7(b,c). Hence, due to the lack of a U-Te-Nd ternary phase diagram, the possibility that U-Te-Nd precipitates analyzed in this study being an undocumented ternary phase cannot be ruled out.

**Table 6:** Crystallographic details from determined phases from SAED patterns presented in Fig. 7(e–g).

Compound	Pearson Symbol	Space Group	Cell Parameters
NdTe^41^	cF8	Fm $\bar{3}$ m (225)	a = *b* = *c* = 0.627 [nm] *α* = *β* = *γ* = 90°
UTe^42^	cF8	Fm $\bar{3}$ m (225)	*a* = *b* = *c* = 0.615 [nm] *α* = *β* = *γ* = 90°
Zr_3_Te^43^	tl32	I $\bar{4}$ (82)	*a* = *b* = 1.134, *c* = 0.563 [nm] *α* = *β* = *γ* = 90°

A medium grey-contrast phase is also found to be present in the HAADF images of Fig. 7. This phase is observed to occupy a large portion of the precipitate phases formed in Fig. 7(b), in addition to forming as a layer surrounding the U-Te-Nd containing precipitates (Fig. 7(b,c)). It also appears to be the primary phase present in the globular precipitate of Fig. 7(d). Analysis of this phase (points 2–4, 8 and 9) indicates that it is a Zr-Te containing phase and based on the atomic ratio of the various points it appears to be the Zr_3_Te compound. Diffraction analysis performed on location *C* (Fig. 7(d)) confirms this phase to be Zr_3_Te. The Zr_3_Te precipitates are observed to contain small white inclusions of varying sizes. EDS analysis of these inclusions observed in both Fig. 7(b,d), point 10, indicates these inclusions are in fact U-Zr precipitates matching that of the U-Zr matrix. Higher magnification of the Zr_3_Te precipitates showed a fine dispersion of such inclusions measuring 10–50 nm in width. The presence of such inclusions would explain why the precipitates appear to be more Zr-rich than the Zr_3_Te compound as well as explain the presence of U contained within the Zr_3_Te precipitates. The middle of the precipitate in Fig. 7(b) contains a dark phase and as indicated by point 5, is found to be a Zr-rich inclusion, similar to those previously observed through SEM analysis (points 7, 8, 15, and 16 in Table 4).

**Figure 8:**
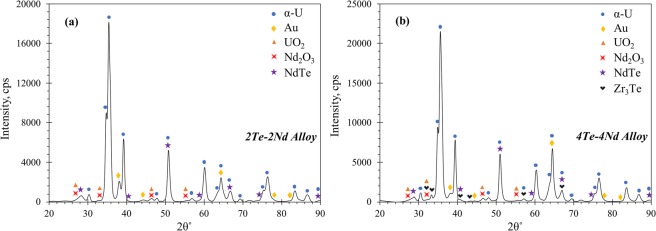
XRD patterns of (**a**) the 2Te-2Nd alloy and (**b**) the 4Te-4Nd alloy.

Focused XRD analysis was also performed on the 2Te-2Nd and 4Te-4Nd alloys, the plots of which are presented in Fig. 8(a,b), respectively. The corresponding crystallographic information of the indexed phases are listed in Table 3. The diffraction patterns for both alloys indicate that each is comprised primarily of the α-U phase. Both alloys also exhibit peaks for the sputtered coating of Au, which was not removed prior to XRD analysis. Both diffraction plots confirm the presence of the NdTe compound, albeit with limited peak intensities. However, such limited intensities would be expected given the compound is only present in the alloy as dispersed precipitates, which were observed to be no larger than 25 μm. As with SAED, no U-Te-Nd ternary phase could be indexed in the XRD patterns. The Zr_3_Te phase is indexed in the XRD pattern of the 4Te-4Nd alloy, further confirming its presence in the alloy. Traces of both UO_2_ and Nd_2_O_3_ were found in both diffraction patterns, and the formation of UO_2_ is, again, likely the result of surface oxidation. The formation of Nd_2_O_3_ can likely be attributed to the combination of surface oxidation and trace amounts of residual oxidation on the Nd feedstock materials that got mixed into the alloy.

In the 2Te-2Nd alloy, all the precipitate phases observed were of NdTe type. This points toward the fact that during the final casting step, when Nd was added to the U-Zr-Te alloy, the existing Zr_2_Te precipitates decomposed, releasing Te to form new precipitates with Nd. The formation of the NdTe compound is favorable, and according to the Nd-Te binary phase diagram it exhibits high temperature stability (*T*_*melt*_ = 2025 °C^28^), which can aid in mitigating availability of Nd to participate in processes leading to FCCI. Characterization of the 4Te-4Nd alloy revealed the formation of several compounds – NdTe and Zr_3_Te as well as a possible U-Te-Nd ternary phase. However, it is important to note that no free Nd was observed in the alloy. This indicates that all available Nd was bound with Te. Therefore, the formation of other Te-based compounds, Zr-Te and U-Te-Nd, would indicate the alloy was Te-rich at this particular analysis plane and the 1:1 additive – lanthanide ratio was not maintained. It is worth recalling that the initial alloys were cast slightly additive-rich and thus the formation of other Te-based compounds beyond Nd-Te could be an indication that less Te may have been lost during casting than what was initially assumed. Regardless, the presence of Nd-Te precipitates demonstrates their propensity to form preferably over Zr-Te compounds.

### Computational prediction of phases

Formation of different compounds observed experimentally as well as the mutual interactions between Te, Nd and Zr can be understood from first principles calculations based on density functional theory (DFT)^16^. DFT calculated enthalpy of mixing (Table 7) for different elements was used as a guidance to predict preferred compound formation tendencies. For each composition in Table 7 the element at the end is the one for which enthalpy of mixing was calculated within a matrix containing all the previous element(s). The enthalpy of mixing of Te inside the α-U matrix is slightly positive, i.e., 0.16 eV, whereas that of Te in the U-Zr system is negative, −0.28 eV. This indicates that Te will bind with the Zr within the U-matrix. Indeed, formation of the Zr_2_Te phase was observed in the U-Zr-Te alloys, as shown in Fig. 1. Further, the enthalpy of mixing of Nd inside the U-Zr matrix is 2.54 eV. This positive enthalpy of mixing indicates a phase-separating tendency of Nd in the U-Zr matrix and thus it is understandable why no Nd containing compound was observed within the U-Zr system. On the other hand, the enthalpy of mixing for Te in the U-Zr-Nd system is −1.48 eV and that in U-Nd is −3.23 eV, suggesting a strong compound forming tendency of Te with Nd regardless of presence or absence of Zr within the U-matrix. With the enthalpy of mixing of Te being more negative in both U-Zr-Nd (−1.48 eV) and U-Nd (−3.23 eV) compared to the enthalpy of mixing of Te in U-Zr (−0.28 eV), the interactions between Nd and Te are expected to be stronger than the interactions between Zr and Te. Thus, it can be concluded that the phase stability in the ternary and in quaternary U-Zr-Nd-Te system can be inferred based on binary interactions between the elements involved (e.g.: Zr-Te interactions vs. Nd-Te interactions).

**Table 7:** Enthalpy of mixing values (as predicted by DFT) for different elements in various systems. A negative enthalpy indicates ease of formation of the stable compound.

Composition	Enthalpy of mixing
U-Te	0.16 eV
U-Zr-Te	−0.28 eV
U-Zr-Nd	2.54 eV
U-Nd-Te	−3.23 eV
U-Zr-Nd-Te	−1.48 eV

**Figure 9:**
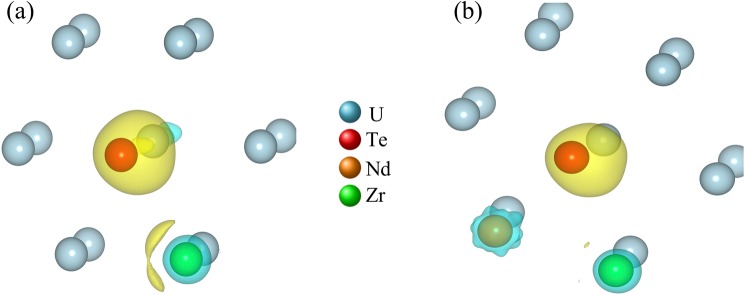
The charge density difference (CDD) between (**a**) U-Zr-Te and U and (**b**) U-Nd-Zr-Te and U systems; the yellow isosurface represents excess charge, and the blue isosurface represents a charge deficit or the negative value of charge density. Note that the figures were created by VESTA 3.0 software, https://onlinelibrary.wiley.com/iucr/doi/10.1107/S0021889811038970.

Furthermore, to understand the interactions between the elements the charge density differences (CDD) have been calculated, as depicted in Fig. 9. The CDD is calculated by taking the difference in the calculated charge densities between final configurations with multiple elements and a pristine U-matrix at each atomic position. From Fig. 9(a), the charge transfer (indicated by a yellow isosurface) observed from Zr to Te indicates a potential electronic interaction between the elements. Such electronic interaction is one of the reasons for the negative enthalpy of mixing that was calculated for Te in the U-Zr matrix. Also, when there is Nd and Te in the system together with Zr, as shown in Fig. 9(b), the charge is noted to transfer from both Nd and Zr towards Te. The excess charge on Te (yellow isosurface) and the charge deficient region on both Nd and Zr (blue isosurface) indicate the attractive interaction of Te with Zr and Nd, which may lead to the formation of a stable compound. A similar interaction was observed between Nd and Te in the system when Zr was absent in the U-matrix^16^.

**Figure 10:**
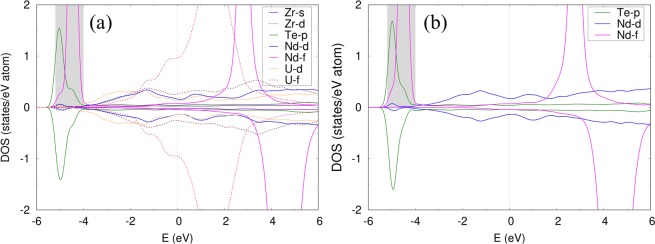
Orbital resolved DOS for (**a**) U-Zr-Te-Nd and (**b**) U-Te-Nd. The vertical dashed lines represent the Fermi level, the energy states up to which states are occupied. The states in the negative and positive vertical axis represent down and up spin components, respectively. U-DOS remain more or less similar in all systems.

To gain a detailed understanding of the interactions between Nd, Te, and Zr, the partial density of states (DOS) have been plotted for Te and Nd in U-Zr-Te-Nd and U-Te-Nd systems, as shown in Fig. 10(a,b), respectively. The occupied 4 *f* states of Nd lies below the Fermi level and is localized between −5 to −4 eV, while the unoccupied 4 *f* states are above the Fermi level. Furthermore, Fig. 10 shows that localized Nd 4 *f* orbitals (solid magenta lines) located between −5 to −4 eV interact with Te (solid green lines) via valence electrons (orbital hybridization), namely, *p* electrons of Te (indicated by the shaded region). The overlapping of the valence orbitals of the additive Te with the Nd *f* orbitals, as shown in Fig. 10(b), was also found to be the key indication for other additives such as Se and As, which were shown to be effective in binding with Nd inside the U-matrix^16^. Hence, from the comparison of both the enthalpy of mixing for Te in U-Zr-Nd and U-Nd matrices as well as the density of states shown in Fig. 10(a,b), it can argued that Te is an effective candidate additive to bind with Nd inside the fuel matrix (U-Zr system).

Results from DFT calculations support the observations made in the characterization of the alloys of interest. The calculations show that the Zr-Te interaction is favorable within the U-Zr-Te alloys. On the other hand, the Nd-Te interactions appear to be even more favorable than Zr-Te, and thus as the fission product Nd forms within the U-Zr-Te alloys, Te is expected to bind with Nd instead of binding with Zr.

## Conclusions

To summarize, both experimental and DFT calculations confirm that binary interactions of Te with both Zr and Nd dominates the phase formations within the multi-component U-Zr-Te and U-Zr-Te-Nd alloys. In the ternary U-Zr-Te alloys, the binary interaction between Te and Zr was observed to be dominant through the formation of Zr_2_Te precipitates. In the quaternary U-Zr-Te-Nd alloys the binary interactions of Te with Nd and Zr were both observed through the formation of NdTe and Zr_3_Te compounds, where the latter phase likely formed due to a Te concentration that exceeded the available Nd. Further, it was concluded that ternary interactions could be plausible through the formation of an unconfirmed U-Te-Nd compound.

It is apparent that Te can be an effective additive to bind with Nd within the U-Zr matrix. Furthermore, the formation of stable, high-melting point, Nd-Te compounds has the potential to stabilize the lanthanide within the fuel, and thus counteract its participation in the FCCI phenomenon. However, further work is necessary to evaluate the stability of the Nd-Te interactions in the non-equilibrium state experienced within an irradiation environment. Additionally, the FCCI phenomenon is driven by diffusion-based mechanisms and therefore further studies on the diffusion interactions of U-Zr-Te-Nd alloys with potential claddings alloys, such as HT-9, are being conducted to further examine the effectiveness of Te as a fuel additive to mitigate FCCI.

### Methodology

#### Experimental methods

Raw materials, such as zirconium, tellurium and neodymium, were acquired from Alfa Aesar company. Neodymium was received as a rod packaged in mylar under argon. Uranium, depleted in the ^235^U isotope to 0.22%, was initially cleaned by submersion in nitric acid, followed by a water wash and a subsequent ethanol wash.

Alloys were cast using an arc-melter, with high purity argon as a cover gas, contained within an argon glovebox. To achieve a homogeneous alloy, the button was melted and flipped three times after each element addition. The U-10Zr-Te alloys were cast using a two-step process, whereas the U-10Zr-Te-Nd alloys were cast using a three-step process. Initially, the uranium and zirconium were added to create a pre-alloy of U-10Zr (wt%). For all four alloys tellurium was added to the U-10Zr buttons in the second step at the appropriate concentration; the button was only flipped and re-melted twice in this step to limit loss of the additive. For the U-10Zr-Te-Nd alloys, neodymium was added to the U-10Zr-Te buttons in the third step at the appropriate concentrations to generate the simulated fuel alloys of interest. The alloys were then drop-cast to form pins with a diameter of approximately 5 mm.

A sample of each alloy pin was sectioned and mounted in a phenolic resin ring and epoxy for microstructural characterization. Samples were prepared by grinding using 320 grit SiC papers to achieve a uniform plane on the sample surface followed by sequential polishing steps with 9 μm, 3 μm and 1 μm polycrystalline diamonds suspended in an ethylene glycol solution. Final polishing steps of the samples were performed within an argon glovebox to limit surface oxidation. The polished surfaces were then sputtered with a 9 nm layer of gold to control charging of the metallographic mount within scanning electron microscope.

Scanning electron microscopy (SEM) was performed using a JEOL JSM-6610LV operated at an accelerating voltage of 20 kV. Imaging of the sample was primarily conducted using the backscatter electron imaging mode (BSE) to provide better contrast of the different phase formations. Energy dispersive x-ray spectroscopy (EDS) was conducted on the SEM in secondary electron (SE) mode using an EDAX Apollo X silicon drift detector (SDD) and the EDAX TEAM v4.4 (2016) software. The *amp time*, which is the time interval that x-ray energy absorption is processed at the detector, was adjusted for each analysis to generate a dead time of approximately 30%. In conducting point analysis, elemental spectra were collected for 50 live seconds per analysis location. In generating elemental X-ray maps a dwell time of 200 μs was used. Elemental data from EDS was quantified based on the standardless ZAF method, which relies on the EDAX TEAM library of stored spectra of standards. Furthermore, it is largely understood that such quantification can have a statistical error of between 2–5%. Transmission electron microscope (TEM) specimens were prepared on a FEI Quanta 3D FEG dual-beam microscope using a focused ion beam (FIB) lift-off technique. The TEM study was conducted using a Tecnai G2 F30 at an accelerating voltage of 300 kV. Both selected area electron diffraction (SAED) patterns and EDS data were collected using bright-field (BF) and high-angle annular dark field (HAADF) detectors. X-ray diffraction (XRD) was performed using a Rigaku SmartLab X-Ray Diffractometer paired with SmartLab Guidance software (v2.0.4.7). The diffractometer was operated at a set tube current of 44 mA using Cu Kα radiation. Analysis was conducted with the optics set in the medium focus, parallel beam (PB) mode paired with a D/tex Ultra 250 1D silicon strip detector. Focused XRD *θ*/2*θ* scans were conducted at several locations on the sample surface using a 0.04° step size and a scan speed of 2°/min. Data was analyzed and indexed using Rigaku’s PDXL 2 software (v2.3.1.0) utilizing the International Centre for Diffraction Data (ICDD) (PDF-2 Release 2016) database for XRD pattern indexing.

### Computational methods

**Figure 11:**
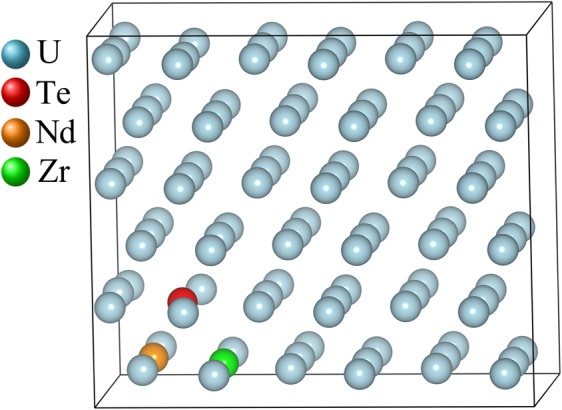
DFT optimized configuration of U-Zr-Te-Nd system. Note that the figure was created by VESTA 3.0 software, https://onlinelibrary.wiley.com/iucr/doi/10.1107/S0021889811038970.

All calculations were performed using the density functional theory (DFT) as implemented in the Vienna *Ab initio* Simulation Package (VASP)^29,30^. Computational details including the choice of *U* for GGA + *U* can be found in the work of Khanal *et al*.^16^. The following procedure was adopted in adding elements within the supercell. First a U atom was substituted with atom A (either Nd, Zr or Te atom) on a fully optimized 3 × 3 × 3 supercell of *α*-U, *Cmcm* (orthorhombic) structure^31^, followed by a re-optimization of the atomic coordinates. After obtaining a newly DFT-optimized atom substituted system, another U-atom at the first nearest neighbor position to atom A was substituted with atom B, and the internal coordinates of the final structure at a fixed volume were optimized again. It is notable that for the quaternary U-Zr-Te-Nd system, several atomic configurations are possible among elements. For finding the most stable configuration of the U-Zr-Te-Nd system, sixteen different possible configurations were created by considering all nearest neighbor non-equivalent arrangements of the third element by fixing two elemental pairs at a time, i.e. Nd-Te, Zr-Te or Nd-Zr. Atomic positions for each configuration were optimized, and the most stable, i.e. minimum energy, configuration among all potential sixteen configurations was chosen for further study. Figure 11 shows an optimized structure of Nd and Te substituted within the U-Zr matrix.

Equation (1) shows a typical equation used to calculate the enthalpy of mixing of an element B in an U-A matrix (Table 7)^32,33^:

1 $$\Delta {E}_{{\rm{m}}{\rm{i}}{\rm{x}}{\rm{i}}{\rm{n}}{\rm{g}}}=E({{\rm{U}}}_{n-2}{\rm{A}}{\rm{B}})+E({\rm{U}})-E({{\rm{U}}}_{n-1}{\rm{A}})-E({\rm{B}}),$$

in which *E*(U_*n*−2_AB) is the total energy of (*n* − 2) U atoms, one A and one B atom in a simulation cell of *n* sites; *E*(U_*n*−1_A) is the total energy of (*n* − 1) U atoms and one A atom in a simulation cell of *n* sites; and *E*(B) and *E*(U) are the total energy per atom for B and U, respectively. Here, negative enthalpy indicates ease of formation of the stable compounds whereas positive enthalpy represents less tendency for the formation of a stable compound. The concept of mixing enthalpy in the formation of intermetallic compounds has been previously used in studying the stability of high entropy alloys (HEA)^34,35^.

## Data Availability

Data generated and analyzed in the current study will be made available upon request to the corresponding author.
